# Psychosis in a Patient with Drug-Resistant Epilepsy: A Case Report

**DOI:** 10.1192/j.eurpsy.2026.11091

**Published:** 2026-07-31

**Authors:** A. Čubranić Dominković, G. Rubeša, I. Ljubičić Bistrović, D. Polić

**Affiliations:** 1 Department of psychiatry, University Hospital Centre Rijeka; 2 Faculty of medicine, University of Rijeka; 3 Department of psychiatry, Teaching Institute of Public Health of Primorsko-Goranska County, Rijeka, Croatia

## Abstract

**Introduction:**

Psychotic episodes in patients with epilepsy present significant diagnostic and therapeutic challenges. The coexistence of epilepsy and psychosis complicates clinical management due to overlapping symptoms, limited antipsychotic options, and risks of drug interactions. This case report describes a 27-year-old patient with long-standing drug-resistant epilepsy who developed a first-time psychotic episode, highlighting complexities in diagnosis and treatment.

**Objectives:**

- To present a rare case of first-time psychosis in a patient with drug-resistant epilepsy.
- To discuss therapeutic challenges related to the selection and management of antipsychotic medication in the context of epilepsy.
- To emphasize diagnostic difficulties when psychosis emerges in epilepsy patient.
- To highlight the importance of multidisciplinary collaboration and a holistic approach in managing complex psychiatric and neurological cases

**Methods:**

Clinical data were obtained from psychiatric evaluations, patient interviews, heteroanamnestic data and medical records during hospitalization. Antiepileptic and antipsychotic therapies were documented and monitored. Observations of clinical response, behavior, and seizure activity were recorded during the inpatient stay.

**Results:**

The patient, a 27-year-old male with epilepsy resistant to treatment since 2009, was hospitalized for a first psychotic episode. He was brought in by police due to aggressive behavior, systematic paranoid delusions, and psychomotor tension. His pre-admission antiepileptic regimen included brivaracetam, topiramate, and clobazam. Quetiapine was initiated and titrated to a total daily dose of 400 mg (100 mg morning, 100 mg afternoon, 200 mg evening). Following this regimen, the patient’s aggression subsided, and no epileptic seizures occurred. However, he displayed dissimulative behavior and social withdrawal from fellow patients. No adverse neurological effects were observed during the treatment.

**Conclusions:**

This case underscores the complexities in managing psychosis in patients with drug-resistant epilepsy. Treatment options are limited by the potential proconvulsant effects of some antipsychotics and the risk of pharmacological interactions. Quetiapine appeared effective and well-tolerated in this patient, but behavioral changes such as social withdrawal warrant close monitoring. The diagnostic process remains challenging, as distinguishing primary psychosis from epilepsy-related psychiatric manifestations requires careful clinical evaluation. Multidisciplinary collaboration and individualized treatment planning are essential for optimizing outcomes in this vulnerable population.

**Disclosure of Interest:**

None Declared

